# Association of *BTLA* Polymorphisms with Susceptibility to Non-Small-Cell Lung Cancer in the Chinese Population

**DOI:** 10.1155/2021/9121824

**Published:** 2021-01-29

**Authors:** Jusi Wang, Zhan Chen, Rui Cao, Qiang Zhang, Tingyu Chen, Chengxiong You, Weifeng Tang, Shuchen Chen

**Affiliations:** ^1^Department of Thoracic Surgery, Fujian Medical University Union Hospital, Fuzhou 350001, China; ^2^Department of Cardiothoracic Surgery, Affiliated People's Hospital of Jiangsu University, Zhenjiang 212000, China

## Abstract

Studies have reported that *B-* and *T-lymphocyte attenuator* (*BTLA*) polymorphisms may be associated with the risk to different cancers. However, the correlation between those variations and non-small-cell lung cancer (NSCLC) is still unclear. A total of 1,003 NSCLC patients and 901 noncancer controls were recruited in the study, to confirm the association of variations in *BTLA* gene with the risk of NSCLC. The SNPscan™ genotyping assay was used to obtain the genotypes of the four BTLA polymorphisms (*BTLA* rs1982809 G>A, rs16859629 T>C, rs2171513 G>A, and rs3112270 A>G). It was found that *BTLA* rs1982809 polymorphism reduced the risk of NSCLC (GA vs. GG: adjusted odds ratio (OR) = 0.81, 95%confidence interval (CI) = 0.66‐0.99, and *P* = 0.043). However, the *BTLA* rs16859629, rs2171513, and rs3112270 polymorphisms showed no significant association between NSCLC patients and controls in overall comparison. In subgroup analyses, we found that *BTLA* rs1982809 polymorphism reduced the risk of NSCLC (nonsquamous cell carcinoma: GA vs. GG: adjusted OR = 0.79, 95%CI = 0.64‐0.97, and *P* = 0.026; AA/GA vs. GG: adjusted OR = 0.81, 95%CI = 0.66‐0.99, and *P* = 0.037; ≥59 years: GA vs. GG: *P* = 0.036; never alcohol consumption: GA vs. GG: *P* = 0.013; GA/AA vs. GG: *P* = 0.016; body mass index (BMI) ≥ 24 kg/m^2^: GA vs. GG: *P* = 0.030; GA/AA vs. GG: *P* = 0.041). The *BTLA* rs16859629 polymorphism increased the risk of the development of squamous cell carcinoma (CC vs. TT: adjusted OR = 9.85, 95%CI = 1.37‐71.03, and *P* = 0.023; CC vs. TT/TC: adjusted OR = 9.55, 95%CI = 1.32‐68.66, and *P* = 0.025). Taken together, the findings of the present suggest that *BTLA* rs1982809 and rs16859629 polymorphisms may influence the susceptibility to NSCLC in the Chinese population.

## 1. Introduction

Non-small-cell lung cancer (NSCLC) accounts for 80 to 85% of all the lung cancer and is the main pathological type of lung cancer (LC). LC has imposed huge diseases burden on human population and accounts for significant number of mortalities across the globe [1–3]. As per estimates, LC is currently ranked as first in terms of incidence and mortality [4]. The currently used treatment strategies for LC include surgery combined with adjuvant therapy. Owing to the recent advancements made in the diagnosis and treatment, the clinical outcomes have significantly improved [5]. However, the identification of the potential risk factors for the occurrence of LC is considered essential as it will permit early diagnosis and immediate management.

The B- and T-lymphocyte attenuator (BTLA), an immunosuppressive receptor, was identified after the cytotoxic T-lymphocyte-associated antigen-4 (CTLA-4) and programmed death-1 (PD-1) and is thus the third member of the CD28 immunoglobulin superfamily (IgSF) [6]. The BTLA upon binding with herpesvirus entry mediator (HVEM) inhibits the T cell response. In contrary, blocking of BTLA may in turn activate T cells [7–9]. Evidences suggest that BTLA plays a crucial immune regulatory role in human malignancies. Liu et al. [10] have reported that BTLA/HVEM pathway plays an important immunosuppressive role in the regulation of T cells in peripheral blood of hepatocellular carcinoma patients. Quan et al. [11] and Li et al. [12] found that the level of BTLA expression may act as a biomarker for the prognosis of diffuse large B cell lymphoma and NSCLC. In yet another study, Chen et al. [13] concluded that chemotherapy in combination with anti-BTLA antibody improves the prognosis of ovarian cancer in a mouse model. Similarly, Zhang et al. [14] reported that BTLA could directly intervene the effects of miR-32 on cancer cells. Collectively, these studies point towards a correlation between BTLA and the development and progression of human cancers.

Single nucleotide polymorphism (SNP) mutations constitute an important form of genetic variations. Some recent studies have revealed a significant correlation between *BTLA* SNPs and development of cancer [15–19]. Fu et al. reported that *BTLA* rs1844089 C>T and rs2705535 A>G variation could increase the risk of breast cancer in human [15]. Partyka et al. and Karabon et al. found that the variation on *BTLA* rs1982809 G>A gene polymorphism might be a potential risk factor for the development of chronic lymphocytic leukemia (CLL) and renal cell carcinoma [16, 17]. In a previous study, we reported that *BTLA* rs1982809 SNPs increased the susceptibility of esophagogastric junction adenocarcinoma (EGJA) in smoking subgroup analyses [18]. In another study, we found that the *BTLA* rs3112270 A>G and rs2171513 G>A polymorphisms could modify the risk of esophageal squamous cell carcinoma (ESCC) [19]. Given this background, we hypothesize that some associations may exist between *BTLA* polymorphisms and NSCLC pathogenesis.

Combined with these previous studies, the *BTLA* tagging SNPs (rs1982809, rs16859629, rs2171513, and rs3112270) were selected for analysis. Consistently, the present study is aimed at exploring the relationship between *BTLA* tagging SNPs and the risk of NSCLC.

## 2. Materials and Methods

### 2.1. Subject

The present study involved 1,003 NSCLC patients and 901 healthy controls. Those patients were continuously recruited from Fujian Medical University Union Hospital, Fuzhou, China, from October 2014 to January 2018, and the diagnosis was confirmed by postoperative pathology. The major inclusion criteria for NSCLC patients were (a) the case firstly to be diagnosed, (b) the individuals with no history of other cancers, (c) without any autoimmune disease, and (d) did not receive any chemo- or radiotherapy prior to enrollment. In the same period, the controls were recruited in the Affiliated Union Hospital of Fujian Medical University (Fuzhou, China) and the Affiliated People's Hospital of Jiangsu University (Zhenjiang, China), and the individuals without any cancer history, mainly healthy, checking individuals, matched with the NSCLC patients by age. The information collected from the patients included age, sex, drinking, smoking history, height, and weight. Furthermore, body mass index (BMI) of more than 24 kg/m^2^ was considered as obesity and overweight [20]. Each participant was informed about the purpose of the study and was asked to sign a written consent form for participation in the study. The study was approved by the research ethics committee of Fujian Medical University Union Hospital (Approval No. 2018KY023).

### 2.2. Selection of *BTLA* Tagging SNPs

The four candidate SNPs were ascertained by applying Genome Variation Server data [18, 19, 21] (http://gvs.gs.washington.edu/GVS147/). We gave priority to represent the main linkage disequilibrium blocks. The following main criteria were used: (a) minor allele frequency ≥ 0.05, (b) linkage disequilibrium of *r*^2^ < 0.8 between the SNPs, (c) with the 5 kb extent upstream and downstream of the gene regions, and (d) the genotyping value ≥ 95% in the CHB cohort. Four BTLA tagging SNPs (rs1982809, rs16859629, rs2171513, and rs3112270) were finally selected to decipher the association between the BTLA polymorphisms and NSCLC risk. The results are shown in Table 1.

### 2.3. DNA Extraction and Genotyping

Around 2 mL of the peripheral blood samples of the subjects (early in the morning, empty stomach) was collected and placed in a vacuum ethylene diamine tetra-acetic acid anticoagulant tube. Genomic DNA was carefully extracted from the collected samples with the help of DNA blood mini kit (Promega, Madison, USA) by following manufacturer's guidelines. The *BTLA* rs1982809, rs16859629, rs2171513, and rs3112270 genotypes were assessed by the SNPscan™ Kit (Genesky Biotechnologies Inc., Shanghai, China) as per manufacturer's guidelines. For the qualitative assessment, 76 samples (4%) were randomly selected and subsequently tested by another laboratory technician. The genotypes of *BTLA* were well confirmed repeatedly.

### 2.4. PCR-Specific Experimental Procedures

#### 2.4.1. DNA Cleavage

The DNA was diluted to a concentration of 30 to 50 ng/*μ*L. From this 4 *μ*L, DNA was transferred to a 96-well plate followed by the addition of 2.5 *μ*L 4X DNA buffer. Thereafter, the total volume was made up to 10 *μ*L by the addition of sterile ddH_2_O. The contents were mixed well and incubated at 98°C for 5 min. Afterwards, the plates were immediately placed in ice.

#### 2.4.2. PCR Reaction

A 10 *μ*L of the premixture prepared was centrifuged at 3000 rpm for 30 seconds. After 4 cycles of the PCR program, the mixture was subjected to 70°C warm bath.

#### 2.4.3. Multiple Fluorescence PCR Reactions

A 19 *μ*L PCR premixture was added to each hole. This was followed thorough mixing and centrifugation at 3000 rpm for 30 seconds. The reaction was subjected to 34 cycles of the PCR program followed by a 4°C warm bath.

#### 2.4.4. Reading of PCR Results

The GeneMapper 4.2 software (Applied Biosystem, USA) was employed for the genotype analysis of the original sequence data.

### 2.5. Statistical Analysis

For the selected *BTLA* genotypes in controls, the internet software (http://ihg.gsf.de/cgi-bin/hw/hwa1.pl) was employed to assess if the genotype frequency distribution conforms to Hardy–Weinberg equilibrium (HWE) or not. Mean ± standard deviation (SD) was adopted for continuous variables. The *t*-test was applied to calculate the difference between NSCLC and healthy subjects. The chi-square (*χ*^2^) or Fisher test was utilized to compare the distributions of the *BTLA* SNPs in related categorical variables between the two groups, including age, gender, tobacco use, drinking, BMI, and genotype frequency. Multivariate logistic regression was used to examine the adjusted odds ratio (OR) and 95% confidence intervals (CIs) in evaluating the relationship between selective *BTLA* SNPs and susceptibility to NSCLC. All data were analyzed by the SAS 9.4 (windows version; SAS Institute Inc., Cary, NC) and SPSS 23 software (windows version; International Business Machines Corp.). Only *P* < 0.05 was considered as a statistically significance.

## 3. Results

### 3.1. Baseline Characteristics

A total of 1,003 NSCLC cases and 901 controls were recruited in this study. The risk factors that may influence the development of NSCLC are listed in Table 2. Although a good match was observed between the age of the NSCLC patients and the control group (*P* = 0.139), significant differences were observed in sex, smoking, alcohol consumption, and BMI between the two groups (*P* < 0.001). The genotyping rate of *BTLA* rs2171513, rs3112270, rs1982809, and rs16859629 was higher than 95% (99.00%, 98.84%, 98.90%, and 97.48%, respectively) as shown in Table 1. Minor allele frequencies (MAFs) in the control were similar to those of the Chinese populations, and the genotype frequency distributions were in accordance with the HWE.

### 3.2. Association between *BTLA* Polymorphisms and Susceptibility to NSCLC

The genotype distribution of *BTLA* rs1982809, rs16859629, rs2171513, and rs3112270 SNPs is shown in Table 3. It was found that GG+AG genotype of *BTLA* rs3112270 polymorphism may be a protective factor for NSCLC compared to the AA genotype (GG+AG: OR = 0.83, 95%CI = 0.69‐0.99, and *P* = 0.038). Meanwhile, compared to the *BTLA* rs1982809 GG genotype, the *BTLA* rs1982809 GA and AA+GA genotypes are also associated with the development of NSCLC (GA: OR = 0.81, 95%CI = 0.67‐0.98, and *P* = 0.030; AA+GA: OR = 0.83, 95%CI = 0.69‐0.99, and *P* = 0.042). Adjusting the risk factors (age, sex, smoking, drinking, and BMI status), the genotypes of *BTLA* rs3112270 polymorphism were not closely correlated with NSCLC susceptibility, whereas the GA genotype of *BTLA* rs1982809 SNPs still decreased the susceptibility of NSCLC patients (GA: adjusted OR = 0.81, 95%CI = 0.66‐0.99, and *P* = 0.043; Table 4).

Collectively, the results suggest that the genotypes in *BTLA* rs2171513 and rs16859629 mutations may be associated with the development of NSCLC.

### 3.3. Association of *BTLA* Polymorphisms with Susceptibility in Different NSCLC Subgroups

The results showed that AA genotype of *BTLA* rs2171513 polymorphism might be considered as a susceptibility factor for the development of squamous cell carcinoma (SCC) (AA/GA: OR = 0.67, 95%CI = 0.45‐0.99, and *P* = 0.044) (Table 4). Adjusting the risk factors (age, sex, smoking, drinking, and BMI status), the *BTLA* rs2171513 polymorphism was not relevant to the occurrence of SCC. Nonetheless, using TT and TT+TC genotypes as references, the results showed that the CC genotype of *BTLA* rs16859629 polymorphisms may promote the development of SCC (CC vs. TT: adjusted OR = 9.85, 95%CI = 1.37‐71.03, and *P* = 0.023; CC vs. TT+TC: adjusted OR = 9.55, 95%CI = 1.32‐68.66, and *P* = 0.025).

In nonsquamous cell carcinoma (non-SCC), using AA genotype as reference, it was found that GG and GA+GG genotypes of *BTLA* rs3112270 polymorphisms may decrease the incidence of non-SCC (AG: OR = 0.82, 95%CI = 0.67‐1.00, and *P* = 0.047; GG+AG: OR = 0.81, 95%CI = 0.67‐0.98, and *P* = 0.033). Meanwhile, it was also found that the GA and GA+AA genotypes of *BTLA* rs1982809 polymorphism also decreased the occurrence of non-SCC with reference to GG genotype (GA vs. GG: OR = 0.79, 95%CI = 0.65‐0.97, and *P* = 0.022; AA/GA vs. GG: OR = 0.81, 95%CI = 0.67‐0.98, and *P* = 0.026). Adjusting the related risk factors, it is concluded that the *BTLA* rs3112270 polymorphism did not exhibit the tendency to change the risk to non-SCC. However, *BTLA* rs1982809 polymorphism was a protective factor of the susceptibility to non-SCC (GA: adjusted OR = 0.79, 95%CI = 0.64‐0.97, and *P* = 0.026; AA+GA: adjusted OR = 0.81, 95%CI = 0.66‐0.99, and *P* = 0.037).

The genotype frequencies of *BTLA* rs1982809 polymorphism in the subgroup analyses are depicted in Table 5. In ≥59-year subgroup, it was found that the variants of *BTLA* rs1982809 decreased the incidence of NSCLC (GA vs. GG: *P* = 0.036). In never alcohol and BMI ≥ 24 kg/m^2^ subgroup, it was found that similar genotype variants of *BTLA* rs1982809 might be a protective factor of NSCLC (never smoking subgroup: GA vs. GG: *P* = 0.013; AA/GA vs. GG: *P* = 0.016; BMI ≥ 24 kg/m^2^: GA vs. GG: *P* = 0.030; AA/GA vs. GG: *P* = 0.041). Additionally, it was found that *BTLA* rs2171513, rs3112270, and rs16859629 polymorphisms were not associated with the morbidity of NSCLC in subgroups (Tables 6–8).

### 3.4. SNP Haplotypes

**Figure 1:**
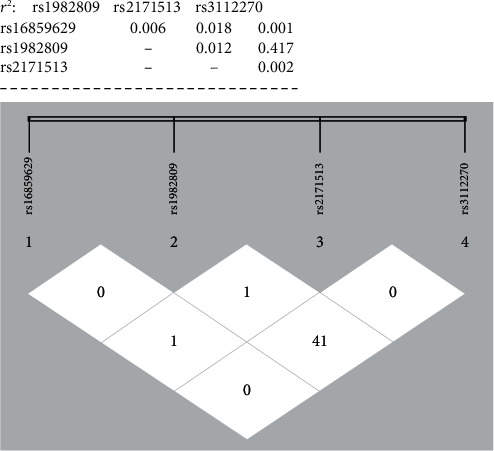
There was no significant linkage disequilibrium between *BTLA* polymorphisms (*r*^2^ < 0.8).

The SHESIS software online (http://analysis.bio-x.cn/myAnalysis.php) was used to perform the haplotype analysis. If the probability of *BTLA* haploid in the case-control study is less than 0.03, it is classified as others. In the end, six subgroups were built. The results are shown in Table 9 and Figure 1. No significant linkage disequilibrium (LD) relationship was observed among the *BTLA* rs16859629, rs1982809, rs2171513, and rs3112270 (*r*^2^ < 0.8). It was found that compared to *BTLA* T_rs16859629_G_rs1982809_G_rs2171513_A_rs3112270_ haplotype, the haplotype *BTLA* T_rs16859629_A_rs1982809_G_rs2171513_G_rs3112270_ significantly reduced the susceptibility to NSCLC (OR = 0.66, 95%CI = 0.659‐0.930, and *P* = 0.005).

## 4. Discussion

The pathogenesis of LC is overly complex. It is believed that LC might be a disease driven by multiple genes [22]. Epidemiological studies have proved the correlation of the etiology of LC and the gene-environment interaction [23, 24]. Recently, immune checkpoint inhibitors have attracted remarkable attention in cancer treatment. For instance, PD-1 and CTLA-4 immunity inhibitors have achieved certain efficacy in the treatment of advanced LC [25]. Nonetheless, the cause of LC is still largely unclear. A previous study had reported that overexpression of BTLA could predict a poor prognosis in NSCLC patients [12].

In the present case-control study, the potential relationship between the *BTLA* rs1982809 G>A, rs16859629 T>C, rs2171513 G>A, and rs3112270 A>G SNPs and susceptibility to NSCLC was explored [23, 26]. It was found that *BTLA* rs1982809 polymorphism might reduce the risk of overall NSCLC. But candidate locus of *BTLA* rs2171513, rs3112270, and rs16859629 SNPs could not affect the susceptibility to NSCLC. In NSCLC subgroup analysis, *BTLA* rs16859629 SNPs could increase the risk of SCC. However, *BTLA* rs1982809 SNPs might reduce susceptibility to non-SCC. In addition, *BTLA* rs1982809 SNPs could reduce the susceptibility to NSCLC in the BMI ≥ 24 kg/m^2^, ≥59 year, and never drinking subgroups. To the best of our knowledge, the present study for the first time reports the relationship between *BTLA* SNPs and NSCLC susceptibility in the Chinese population.

BTLA rs1982809, as a locus in the 3′-untranslated region (UTR), has been reported to exhibit an association with the development of some malignancies. The 3′-UTR plays a crucial role in the regulation of mRNA expression [27, 28]. Karabon et al. reported that *BTLA* could exert its effects on T-lymphocytes by affecting mRNA expression levels due to T to C substitutions of rs1982809 [16]. Additionally, it was also reported that BTLA rs1982809 might act as a potential biomarker in predicting the multiple organ dysfunction syndrome. In another study, rs1982809 polymorphism of the *BTLA* was found to be associated with the risk of kidney cancer [17]. Similarly, in our previous study, we report that the gene variation in *BTLA* rs1982809 increased the susceptibility to EGJA in ever smoking subjects [18]. In contrary, Cao et al. concluded that the distribution of genotype in *BTLA* rs1982809 was not different from the ESCC and the control group [19]. In short, the previous results were ambiguous, even contradictory. Considering that genetic variations played different roles in different cancers, 1,904 participants were enrolled to conduct a more precise evaluation. In this study, we showed that the G to A changes of BTLA rs1982809 genotype reduced the overall risk of NSCLC, especially the non-SCC, BMI ≥ 24 kg/m^2^, ≥59 year, and never drinking subgroups. These facts indicated that *BTLA* rs1982809 polymorphism could play a critical role in the susceptibility to NSCLC. However, our findings should be interpreted with cautions. Studies with larger sample sizes are required to further validate the effects of this locus on NSCLC.

The *BTLA* rs16859629 SNP, which is located in the intron variant sequence, plays an important role in alternative splicing [29]. It has been reported that SNP located in the intron regions may affect the susceptibility to several human diseases [30–32]. When adjusted for including 5 covariates by regression analysis, it was found that *BTLA* rs16859629 SNP increased the risk of SCC. In two recent studies, the genotype distribution of *BTLA* rs16859629 showed no statistically significant differences between EGJA cases and controls and the same conclusion was made for susceptibility to ESCC [18, 19]. Interestingly, the present study first explored that *BTLA* rs16859629 SNP could promote the susceptibility to cancer. However, considering that the number of SCC was relatively small, the results might not be convincing enough. In the future, a larger case-control group study should be performed to unveil the susceptibility relationship between *BTLA* rs16859629 and SCC.

Previous studies have shown that the genetics of the SNP locus in the *BTLA* rs16859629, rs1982809, rs2171513, and rs3112270 may not be random [18]. In the present study, it was found that haploid type of the four candidate SNP loci may play an important role in heredity. It was found that the haplotype *BTLA* T_rs16859629_A_rs1982809_G_rs2171513_G_rs3112270_ could significantly change the susceptibility to NSCLC, affecting 18.70% of the normal population. Interestingly, compared to the study by Tang et al. [18], it was found that with alteration of rs1982809, the effect of this single locus on haplotype significantly reversed the association with cancer. When *BTLA* rs1982809 was allele A, it decreased the risk of NSCLC. This is in contrary to the findings of Tang et al. [18]. However, our findings need to be further verified in the future studies. In addition, stepwise analysis of the SPSS 23 software was used to analyze the correlation of age, sex, smoking, drinking, BMI, and the four candidate SNPs. We found that the *BTLA* rs1982809 among the four candidate SNPs may be a key factor affecting the susceptibility to NSCLC. The detailed results are shown in supplementary document 1.

Despite some interesting findings, our study suffers from the following potential limitations. Firstly, our sample is only from two hospitals, the Affiliated Hospital of Fujian Medical University and Jiangsu University. Although the four selected SNPs were consistent with HWE and there is no significant difference between MAF and database of Chinese Han populations, the bias might still be unavoidable. Secondly, the sample sizes of some subgroups are relatively small, especially in the SCC subgroup. As such, our conclusions may not be sufficient to testify the real relationship of *BTLA* polymorphisms with susceptibility to SCC. The replication of the study with larger sample sizes is required to further validate our findings. Thirdly, only four functional loci in *BTLA* gene were selected in our study, and, therefore, other polymorphisms of *BTLA* should not be ignored. Finally, a functional study for these identified SNPs was not performed.

## 5. Conclusions

Despite some shortcomings, our findings preliminarily suggest that *BTLA* rs1982809 and rs16859629 SNPs may contribute to the risk of NSCLC. However, a thorough study with larger samples should be worth to elucidate the potential molecular functions of these *BTLA* polymorphisms.

## Figures and Tables

**Table 1:** Primary information for *BTLA* tagging polymorphisms.

Genotyped polymorphisms	rs2171513 G>A	rs3112270 A>G	rs1982809 G>A	rs16859629 T>C
Chr	3	3	3	3
Position_38	112466080	112461797	112463893	112471533
Region	3′-UTR	Promoter	3′-UTR	Intron_variant
MAF in database (1000 genomes—Chinese Han populations)	0.198	0.278	0.195	0.082
MAF in our controls (*n* = 901)	0.194	0.295	0.270	0.079
*P* value for HWE test in our controls	0.554	0.259	0.584	0.747
% genotyping value	99.00%	98.84%	98.90%	97.48%

Abbreviations: MAF: minor allele frequency; HWE: Hardy–Weinberg equilibrium.

**Table 2:** Distribution of selected demographic variables and risk factors in NSCLC cases and controls.

Variable	Cases (*n* = 1,003)		Controls (*n* = 901)		*P* ^a^
	*n*	%	*n*	%	
Age (years)	58.76 ± 9.92		59.43 ± 9.67		0.139
Age (years)					0.292
<59	465	46.36	396	43.95	
≥59	538	53.64	505	56.05	
Sex					<0.001
Male	520	51.84	552	61.27	
Female	483	48.16	349	38.73	
Tobacco use					<0.001
Never	634	63.21	717	79.58	
Ever	369	36.79	184	20.42	
Alcohol use					<0.001
Never	789	78.66	804	89.23	
Ever	214	21.34	97	10.77	
BMI (kg/m^2^)					<0.001
<24	672	67.00	497	55.16	
≥24	331	33.00	404	44.84	
Type of NSCLC					
SCC	145	14.46			
Non-SCC	858	85.54			

Notes: ^a^two-sided *χ*^2^ test and Student's *t*-test. Abbreviations: BMI: body mass index; NSCLC: non-small-cell lung cancer; SCC: squamous cell carcinoma.

**Table 3:** The frequencies of *BTLA* tagging polymorphisms in different NSCLC subgroups.

Genotype	NSCLC (*n* = 1,003)		SCC (*n* = 145)		Non-SCC (*n* = 858)		Controls (*n* = 901)	
	*n*	%	*n*	%	*n*	%	*n*	%
rs2171513 G>A								
GG	654	66.06	106	73.61	548	64.78	582	65.03
GA	297	30.00	35	24.31	262	30.97	276	30.84
AA	39	3.94	3	2.08	36	4.26	37	4.13
A allele	375	18.94	41	14.24	334	19.74	350	19.55
rs3112270 A>G								
AA	527	53.39	74	51.39	453	53.74	435	48.60
AG	392	39.72	61	42.36	331	39.26	388	43.35
GG	68	6.89	9	6.25	59	7.00	72	8.04
G allele	528	26.75	79	27.43	449	26.63	532	29.72
rs1982809 G>A								
GG	566	57.29	77	53.47	489	57.94	471	52.63
GA	351	35.53	54	37.50	297	35.19	361	40.34
AA	71	7.19	13	9.03	58	6.87	63	7.04
A allele	493	24.95	80	27.78	413	24.47	487	27.21
rs16859629 T>C								
TT	835	85.82	115	82.14	720	86.43	746	84.48
TC	131	13.46	23	16.43	108	12.97	132	14.95
CC	7	0.72	2	1.43	5	0.60	5	0.57
C allele	145	7.45	27	9.64	118	7.08	142	8.04

**Table 4:** Logistic regression analyses of association of *BTLA* tagging polymorphisms with risk of NSCLC.

Genotype	Overall NSCLC cases (*n* = 1,003) vs. controls (*n* = 901)				SCC (*n* = 145) vs. controls (*n* = 901)				Non-SCC (*n* = 858) vs. controls (*n* = 901)			
	Crude OR (95% CI)	*P*	Adjusted OR^a^ (95% CI)	*P*	Crude OR (95% CI)	*P*	Adjusted OR^a^ (95% CI)	*P*	Crude OR (95% CI)	*P*	Adjusted OR^a^ (95% CI)	*P*
rs2171513 G>A												
GA vs. GG	0.96 (0.79-1.17)	0.669	1.00 (0.81-1.24)	0.971	0.70 (0.46-1.05)	0.082	0.83 (0.51-1.32)	0.426	1.01 (0.82-1.24)	0.938	1.03 (0.83-1.28)	0.760
AA vs. GG	0.94 (0.59-1.49)	0.787	0.97 (0.60-1.59)	0.908	0.45 (0.14-1.47)	0.184	0.32 (0.09-1.18)	0.086	1.03 (0.64-1.66)	0.892	1.06 (0.65-1.74)	0.816
AA/GA vs. GG	0.96 (0.79-1.16)	0.637	1.00 (0.82-1.22)	1.00	0.67 (0.45-0.99)	0.044	0.74 (0.47-1.17)	0.195	1.01 (0.83-1.23)	0.912	1.04 (0.84-1.28)	0.728
AA vs. GG/GA	0.95 (0.60-1.51)	0.830	0.97 (0.60-1.58)	0.904	0.49 (0.15-1.62)	0.245	0.34 (0.09-1.23)	0.099	1.03 (0.65-1.65)	0.900	1.05 (0.64-1.72)	0.847
rs3112270 A>G												
AG vs. AA	0.83 (0.69-1.01)	0.060	0.86 (0.70-1.05)	0.125	0.92 (0.64-1.33)	0.672	1.10 (0.72-1.69)	0.667	0.82 (0.67-1.00)	0.047	0.82 (0.67-1.01)	0.064
GG vs. AA	0.78 (0.55-1.11)	0.169	0.81 (0.56-1.18)	0.275	0.74 (0.35-1.53)	0.412	0.80 (0.35-1.85)	0.603	0.79 (0.54-1.14)	0.202	0.81 (0.55-1.20)	0.293
GG/AG vs. AA	0.83 (0.69-0.99)	0.038	0.85 (0.70-1.03)	0.092	0.90 (0.63-1.27)	0.535	1.05 (0.70-1.58)	0.818	0.81 (0.67-0.98)	0.033	0.82 (0.67-1.00)	0.051
GG vs. AA/AG	0.85 (0.60-1.19)	0.340	0.87 (0.61-1.25)	0.456	0.76 (0.37-1.56)	0.458	0.77 (0.34-1.73)	0.522	0.86 (0.60-1.23)	0.411	0.89 (0.61-1.29)	0.530
rs1982809 G>A												
GA vs. GG	0.81 (0.67-0.98)	0.030	0.81 (0.66-0.99)	0.043	0.92 (0.63-1.33)	0.642	1.05 (0.68-1.62)	0.833	0.79 (0.65-0.97)	0.022	0.79 (0.64-0.97)	0.026
AA vs. GG	0.94 (0.65-1.35)	0.727	0.99 (0.68-1.45)	0.960	1.26 (0.66-2.40)	0.478	1.83 (0.84-3.97)	0.130	0.89 (0.61-1.30)	0.534	0.93 (0.63-1.39)	0.724
AA/GA vs. GG	0.83 (0.69-0.99)	0.042	0.84 (0.69-1.02)	0.071	0.97 (0.68-1.38)	0.850	1.15 (0.76-1.73)	0.522	0.81 (0.67-0.98)	0.026	0.81 (0.66-0.99)	0.037
AA vs. GG/GA	1.02 (0.72-1.45)	0.901	1.08 (0.74-1.57)	0.690	1.31 (0.70-2.45)	0.396	1.79 (0.84-3.80)	0.131	0.98 (0.67-1.41)	0.891	1.03 (0.70-1.51)	0.900
rs16859629 T>C												
TC vs. TT	0.89 (0.68-1.15)	0.367	0.89 (0.68-1.18)	0.412	1.13 (0.70-1.84)	0.620	1.19 (0.68-2.11)	0.544	0.85 (0.64-1.12)	0.238	0.86 (0.65-1.15)	0.307
CC vs. TT	1.25 (0.40-3.96)	0.703	1.38 (0.41-4.58)	0.602	2.60 (0.50-13.53)	0.258	9.85 (1.37-71.03)	0.023	1.04 (0.30-3.59)	0.955	1.09 (0.30-4.01)	0.898
CC/TC vs. TT	0.90 (0.70-1.16)	0.419	0.91 (0.69-1.19)	0.486	1.18 (0.74-1.89)	0.481	1.31 (0.75-2.28)	0.338	0.86 (0.65-1.12)	0.253	0.87 (0.65-1.15)	0.330
CC vs. TT/TC	1.27 (0.40-4.02)	0.682	1.40 (0.42-4.66)	0.584	2.55 (0.49-13.25)	0.267	9.55 (1.32-68.66)	0.025	1.06 (0.31-3.68)	0.926	1.11 (0.30-4.09)	0.873

Notes: ^a^adjusted for age, sex, smoking status, alcohol use, and BMI status in a logistic regression model.

**Table 5:** Stratified analyses between *BTLA* rs1982809 G>A polymorphism and NSCLC risk by sex, age, smoking status, alcohol consumption, and BMI.

Variable	*BTLA* rs1982809 G>A (case/control)^a^			Adjusted OR^b^ (95% CI); *P*				
	GG	GA	AA	GG	GA vs. GG	AA vs. GG	GA/AA vs. GG	AA vs. (GG/GA)
Sex								
Male	299/289	177/228	37/33	1.00	0.79 (0.60-1.05); *P*: 0.100	1.32 (0.77-2.27); *P*: 0.314	0.86 (0.66-1.11); *P*: 0.246	1.45 (0.86-2.46); *P*: 0.167
Female	267/182	174/133	34/30	1.00	0.87 (0.64-1.18); *P*: 0.366	0.77 (0.45-1.33); *P*: 0.352	0.85 (0.64-1.14); *P*: 0.274	0.82 (0.49-1.38); *P*: 0.457
Age								
<59	254/203	173/162	31/28	1.00	0.93 (0.69-1.27); *P*: 0.651	0.90 (0.50-1.62); *P*: 0.728	0.93 (0.69-1.24); *P*: 0.611	0.93 (0.52-1.64); *P*: 0.799
≥59	312/268	178/199	40/35	1.00	0.75 (0.57-0.98); *P*: 0.036	1.08 (0.65-1.78); *P*: 0.778	0.79 (0.61-1.03); *P*: 0.079	1.21 (0.74-1.98); *P*: 0.450
Smoking status								
Never	355/371	220/285	49/55	1.00	0.79 (0.62-1.01); *P*: 0.060	0.91 (0.59-1.40); *P*: 0.651	0.81 (0.65-1.02); *P*: 0.074	1.00 (0.65-1.52); *P*: 0.980
Ever	211/100	131/76	22/8	1.00	0.86 (0.59-1.26); *P*: 0.444	1.32 (0.56-3.11); *P*: 0.525	0.91 (0.63-1.31); *P*: 0.603	1.40 (0.60-3.26); *P*: 0.433
Alcohol consumption								
Never	443/408	280/332	55/59	1.00	0.76 (0.61-0.94); *P*: 0.013	0.87 (0.58-1.31); *P*: 0.509	0.77 (0.63-0.95); *P*: 0.016	0.98 (0.66-1.46); *P*: 0.911
Ever	123/63	71/29	16/4	1.00	1.22 (0.70-2.14); *P*: 0.479	2.55 (0.75-8.60); *P*: 0.132	1.36 (0.80-2.32); *P*: 0.256	2.37 (0.71-7.86); *P*: 0.159
BMI (kg/m^2^)								
<24	368/257	245/201	47/35	1.00	0.89 (0.68-1.15); *P*: 0.361	1.06 (0.65-1.73); *P*: 0.828	0.91 (0.71-1.17); *P*: 0.458	1.11 (0.69-1.80); *P*: 0.667
≥24	198/214	106/160	24/28	1.00	0.69 (0.50-0.97); *P*: 0.030	0.88 (0.48-1.62); *P*: 0.677	0.72 (0.53-0.99); *P*: 0.041	1.01 (0.56-1.84); *P*: 0.973

Notes: ^a^the genotyping was successful in 1,003 (98.50%) NSCLC cases, and 901 (99.33%) controls for *BTLA* rs1982809 G>A. ^b^Adjusted for age, sex, smoking status, alcohol consumption, and BMI (besides stratified factors accordingly) in a logistic regression model. Abbreviations: BMI: body mass index; NSCLC: non-small-cell lung cancer.

**Table 6:** Stratified analyses between *BTLA* rs2171513 G>A polymorphism and NSCLC risk by sex, age, smoking status, alcohol consumption, and BMI.

Variable	*BTLA* rs2171513 G>A (case/control)^a^			Adjusted OR^b^ (95% CI); *P*				
	GG	GA	AA	GG	GA vs. GG	AA vs. GG	GA/AA vs. GG	AA vs. (GG/GA)
Sex								
Male	335/361	165/165	15/24	1.00	1.15 (0.86-1.53); *P*: 0.346	0.70 (0.34-1.43); *P*: 0.325	1.09 (0.83-1.44); *P*: 0.545	0.67 (0.33-1.36); *P*: 0.266
Female	319/221	132/111	24/13	1.00	0.84 (0.61-1.15); *P*: 0.273	1.32 (0.65-2.69); *P*: 0.441	0.89 (0.66-1.20); *P*: 0.445	1.40 (0.69-2.83); *P*: 0.350
Age								
<59	305/265	133/114	21/14	1.00	1.02 (0.73-1.40); *P*: 0.930	1.58 (0.73-3.42); *P*: 0.244	1.06 (0.78-1.46); *P*: 0.672	1.58 (0.73-3.39); *P*: 0.244
≥59	349/317	164/162	18/23	1.00	0.98 (0.74-1.30); *P*: 0.906	0.67 (0.35-1.30); *P*: 0.240	0.94 (0.72-1.23); *P*: 0.659	0.68 (0.35-1.30); *P*: 0.242
Smoking status								
Never	408/456	188/227	29/28	1.00	0.97 (0.76-1.24); *P*: 0.803	1.18 (0.67-2.08); *P*: 0.571	0.99 (0.78-1.26); *P*: 0.948	1.19 (0.68-2.09); *P*: 0.543
Ever	246/126	109/49	10/9	1.00	1.08 (0.72-1.63); *P*: 0.698	0.58 (0.23-1.48); *P*: 0.254	1.01 (0.68-1.48); *P*: 0.980	0.57 (0.22-1.44); *P*: 0.232
Alcohol consumption								
Never	520/520	226/245	34/34	1.00	0.98 (0.78-1.23); *P*: 0.829	0.99 (0.59-1.66); *P*: 0.967	0.98 (0.79-1.22); *P*: 0.834	1.00 (0.60-1.66); *P*: 0.990
Ever	134/62	71/31	5/3	1.00	1.13 (0.65-1.95); *P*: 0.670	0.79 (0.17-3.69); *P*: 0.767	1.10 (0.64-1.87); *P*: 0.735	0.76 (0.17-3.50); *P*: 0.725
BMI (kg/m^2^)								
<24	441/326	194/147	26/20	1.00	1.05 (0.80-1.38); *P*: 0.711	0.91 (0.49-1.69); *P*: 0.761	1.03 (0.80-1.34); *P*: 0.803	0.89 (0.48-1.66); *P*: 0.724
≥24	213/256	103/129	13/17	1.00	0.93 (0.66-1.30); *P*: 0.656	1.09 (0.49-2.40); *P*: 0.839	0.94 (0.68-1.30); *P*: 0.723	1.11 (0.51-2.44); *P*: 0.791

Notes: ^a^the genotyping was successful in 1,003 (98.70%) NSCLC cases, and 901 (99.33%) controls for *BTLA* rs2171513 G>A. ^b^Adjusted for age, sex, smoking status, alcohol consumption, and BMI (besides stratified factors accordingly) in a logistic regression model. Abbreviations: BMI: body mass index; NSCLC: non-small-cell lung cancer.

**Table 7:** Stratified analyses between *BTLA* rs3112270 A>G polymorphism and NSCLC risk by sex, age, smoking status, alcohol consumption, and BMI.

Variable	*BTLA* rs3112270 A>G (case/control)^a^			Adjusted OR^b^ (95% CI); *P*				
	AA	GA	GG	AA	AG vs. AA	GG vs. AA	AG/GG vs. AA	GG vs. (AA/AG)
Sex								
Male	277/270	199/237	37/43	1.00	0.89 (0.68-1.17); *P*: 0.413	0.97 (0.58-1.62); *P*: 0.902	0.90 (0.69-1.18); *P*: 0.447	1.02 (0.62-1.68); *P*: 0.940
Female	250/165	193/151	31/29	1.00	0.84 (0.62-1.13); *P*: 0.257	0.72 (0.41-1.26); *P*: 0.250	0.82 (0.62-1.09); *P*: 0.179	0.78 (0.45-1.34); *P*: 0.367
Age								
<59	243/195	184/163	31/35	1.00	0.92 (0.68-1.26); *P*: 0.615	0.81 (0.47-1.43); *P*: 0.472	0.91 (0.68-1.21); *P*: 0.507	0.84 (0.49-1.45); *P*: 0.539
≥59	284/240	208/225	37/37	1.00	0.82 (0.63-1.07); *P*: 0.140	0.84 (0.50-1.39); *P*: 0.493	0.82 (0.63-1.06); *P*: 0.131	0.92 (0.56-1.51); *P*: 0.733
Smoking status								
Never	330/339	246/316	47/56	1.00	0.81 (0.64-1.03); *P*: 0.081	0.87 (0.56-1.35); *P*: 0.528	0.82 (0.65-1.03); *P*: 0.084	0.96 (0.63-1.46); *P*: 0.836
Ever	197/96	146/72	21/16	1.00	1.01 (0.69-1.47); *P*: 0.980	0.67 (0.33-1.37); *P*: 0.278	0.94 (0.66-1.36); *P*: 0.758	0.67 (0.34-1.35); *P*: 0.263
Alcohol consumption								
Never	410/383	312/350	56/66	1.00	0.85 (0.69-1.06); *P*: 0.156	0.78 (0.52-1.17); *P*: 0.230	0.84 (0.69-1.04); *P*: 0.106	0.84 (0.57-1.24); *P*: 0.380
Ever	117/52	80/38	12/6	1.00	0.89 (0.52-1.52); *P*: 0.666	1.14 (0.38-3.46); *P*: 0.818	0.92 (0.55-1.54); *P*: 0.747	1.20 (0.41-3.55); *P*: 0.742
BMI (kg/m^2^)								
<24	355/233	255/217	49/43	1.00	0.79 (0.61-1.02); *P*: 0.070	0.81 (0.51-1.29); *P*: 0.369	0.79 (0.62-1.01); *P*: 0.063	0.90 (0.57-1.41); *P*: 0.646
≥24	172/202	137/171	19/29	1.00	0.96 (0.70-1.32); *P*: 0.787	0.82 (0.34-1.55); *P*: 0.543	0.94 (0.69-1.27); *P*: 0.677	0.84 (0.45-1.56); *P*: 0.574

Notes: ^a^the genotyping was successful in 1,003 (98.40%) NSCLC cases, and 901 (99.33%) controls for *BTLA* rs3112270 A>G; ^b^adjusted for age, sex, smoking status, alcohol consumption, and BMI (besides stratified factors accordingly) in a logistic regression model. Abbreviations: BMI: body mass index; NSCLC: non-small-cell lung cancer.

**Table 8:** Stratified analyses between *BTLA* rs16859629 T>C polymorphism and NSCLC risk by sex, age, smoking status, and alcohol consumption.

Variable	*BTLA* rs16859629 T>C (case/control)^a^			Adjusted OR^b^ (95% CI); *P*				
	TT	TC	CC	TT	TC vs. TT	CC vs. TT	TC/CC vs. TT	CC vs. (TT/TC)
Sex								
Male	435/459	70/81	3/1	1.00	0.91 (0.63-1.33); *P*: 0.636	5.89 (0.56-61.95); *P*: 0.140	0.96 (0.66-1.39); *P*: 0.815	5.97 (0.57-62.73); *P*: 0.137
Female	400/287	61/51	4/4	1.00	0.85 (0.57-1.29); *P*: 0.455	0.70 (0.17-2.89); *P*: 0.618	0.84 (0.57-1.26); *P*: 0.402	0.71 (0.17-2.95); *P*: 0.640
Age								
<59	387/325	58/58	4/3	1.00	0.80 (0.52-1.22); *P*: 0.303	1.17 (0.24-5.76); *P*: 0.848	0.82 (0.54-1.24); *P*: 0.342	1.21 (0.45-5.93); *P*: 0.819
≥59	448/421	73/74	3/2	1.00	0.96 (0.67-1.39); *P*: 0.843	1.56 (0.24-10.30); *P*: 0.643	0.98 (0.68-1.41); *P*: 0.908	1.57 (0.24-10.35); *P*: 0.639
Smoking status								
Never	527/593	80/107	6/5	1.00	0.85 (0.61-1.18); *P*: 0.325	1.21 (0.35-4.19); *P*: 0.766	0.87 (0.63-1.19); *P*: 0.376	1.24 (0.36-4.29); *P*: 0.738
Ever	308/153	51/25	1/0	1.00	1.00 (0.59-1.70); *P*: 0.988	-	1.02 (0.60-1.72); *P*: 0.945	-
Alcohol consumption								
Never	659/665	102/117	6/5	1.00	0.89 (0.66-1.21); *P*: 0.457	1.24 (0.36-4.31); *P*: 0.734	0.91 (0.68-1.22); *P*: 0.513	1.26 (0.36-4.37); *P*: 0.715
Ever	176/81	29/15	1/0	1.00	0.86 (0.42-1.75); *P*: 0.681	-	0.89 (0.44-1.80); *P*: 0.735	-
BMI (kg/m^2^)								
<24	557/411	88/71	4/2	1.00	0.92 (0.65-1.32); *P*: 0.658	1.81 (0.30-10.83); *P*: 0.514	0.95 (0.67-1.34); *P*: 0.752	1.84 (0.31-10.95); *P*: 0.505
≥24	278/335	43/61	3/3	1.00	0.83 (0.53-1.29); *P*: 0.411	1.08 (0.20-5.86); *P*: 0.930	0.84 (0.55-1.30); *P*: 0.437	1.11 (0.20-6.00); *P*: 0.906

Notes: ^a^the genotyping was successful in 1,003 (97.01%) NSCLC cases, and 901 (98.00%) controls for *BTLA* rs16859629 T>C; ^b^adjusted for age, sex, smoking status, alcohol consumption, and BMI (besides stratified factors accordingly) in a logistic regression model. Abbreviations: BMI: body mass index; NSCLC: non-small-cell lung cancer.

**Table 9:** *BTLA* haplotype analysis.

Haplotypes	Case (*n* = 2006)		Control (*n* = 1802)		Crude OR (95% CI)	*P*
	*n*	%	*n*	%		
T G G A	1015	52.40	854	48.40	1.00	
T A G G	295	15.30	330	18.70	0.661 (0.659-0.930)	0.005
T G A A	207	10.70	189	10.70	0.843 (0.810-1.229)	0.982
T G A G	100	5.20	102	5.80	0.757 (0.674-1.189)	0.446
C G G A	80	4.10	92	5.20	0.662 (0.577-1.066)	0.119
T A G A	63	3.30	47	2.70	1.034 (0.836-1.794)	0.298
T G G G	49	2.50	48	2.70	0.919 (0.614-1.374)	0.680
T A A A	42	2.20	48	2.70	0.674 (0.525-1.213)	0.291
C A G G	47	2.40	37	2.10	0.972 (0.745-1.779)	0.526
Others	37	1.90	17	0.90	3.595 (1.502-12.065)	<0.003

The haplotypes were constructed in the sequence of BTLA rs16859629 T>C, rs1982809 G>A, rs2171513 G>A, and rs3112270 A>G.

## Data Availability

The data used to support the findings of this study are available from the corresponding author according to reasonable request.

## Supplementary Materials

Supplementary Materials Please see the document 1 about stepwise analysis of BTLA rs16859629, rs1982809, rs2171513, and rs3112270.
