# Supplementary material for: Association of BTLA Polymorphisms with Susceptibility to Non-Small-Cell Lung Cancer in the Chinese Population
Source: Biomed Res Int. 2021 Jan 29;2021:9121824. doi: 10.1155/2021/9121824 (PMC7867466; doi:10.1155/2021/9121824)
Supplement: Supplementary Materials — Please see the document 1 about stepwise analysis of BTLA rs16859629, rs1982809, rs2171513, and rs3112270. [file 9121824.f1.doc]

**The univariate analysis**

**Table 1a BTLA rs1982809**

| Variables in the Equation | | | | | | | | | |
| --- | --- | --- | --- | --- | --- | --- | --- | --- | --- |
|  | | B | S.E. | Wald | df | Sig. | Exp(B) | 95% C.I.for EXP(B) | |
|  |  |  |  |  |  |  |  | Lower | Upper |
| Step 1^a^ | sex(1) | 1.265 | .122 | 107.820 | 1 | .000 | 3.544 | 2.791 | 4.500 |
|  | age | .010 | .005 | 3.716 | 1 | .054 | 1.010 | 1.000 | 1.020 |
|  | smoking(1) | -1.340 | .137 | 95.382 | 1 | .000 | .262 | .200 | .343 |
|  | drinking(1) | -.744 | .154 | 23.179 | 1 | .000 | .475 | .351 | .643 |
|  | BMI(1) | .476 | .100 | 22.567 | 1 | .000 | 1.609 | 1.322 | 1.958 |
|  | rs1982809 |  |  | 4.756 | 4 | .313 |  |  |  |
|  | rs1982809(1) | -.141 | .321 | .192 | 1 | .661 | .869 | .463 | 1.630 |
|  | rs1982809(2) | -21.522 | 27844.094 | .000 | 1 | .999 | .000 | .000 | . |
|  | rs1982809(3) | .004 | .197 | .000 | 1 | .982 | 1.004 | .683 | 1.477 |
|  | rs1982809(4) | .211 | .104 | 4.088 | 1 | .043 | 1.235 | 1.006 | 1.515 |
|  | Constant | -1.136 | .311 | 13.343 | 1 | .000 | .321 |  |  |
| a. Variable(s) entered on step 1: sex, age, smoking, drinking, BMI, rs1982809. | | | | | | | | | |

**Table 1b BTLA rs1982809**

| Model if Term Removed^a^ | | | | | |
| --- | --- | --- | --- | --- | --- |
| Variable | | Model Log Likelihood | Change in -2 Log Likelihood | df | Sig. of the Change |
| Step 1 | sex | -1262.568 | 115.674 | 1 | .000 |
|  | age | -1206.596 | 3.729 | 1 | .053 |
|  | smoking | -1254.564 | 99.665 | 1 | .000 |
|  | drinking | -1216.542 | 23.621 | 1 | .000 |
|  | BMI | -1216.088 | 22.713 | 1 | .000 |
|  | **rs1982809** | **-1208.920** | **8.376** | **4** | **.079** |
| a. Based on conditional parameter estimates | | | | | |

**Table 2a BTLA rs16859629**

| Variables in the Equation | | | | | | | | | |
| --- | --- | --- | --- | --- | --- | --- | --- | --- | --- |
|  | | B | S.E. | Wald | df | Sig. | Exp(B) | 95% C.I.for EXP(B) | |
|  |  |  |  |  |  |  |  | Lower | Upper |
| Step 1^a^ | sex(1) | 1.256 | .122 | 106.777 | 1 | .000 | 3.512 | 2.767 | 4.457 |
|  | age | .009 | .005 | 3.335 | 1 | .068 | 1.009 | .999 | 1.019 |
|  | smoking(1) | -1.333 | .137 | 94.922 | 1 | .000 | .264 | .202 | .345 |
|  | drinking(1) | -.751 | .154 | 23.722 | 1 | .000 | .472 | .349 | .638 |
|  | BMI(1) | .470 | .100 | 22.110 | 1 | .000 | 1.600 | 1.315 | 1.946 |
|  | rs16859629 |  |  | 1.368 | 3 | .713 |  |  |  |
|  | rs16859629(1) | -.207 | .319 | .420 | 1 | .517 | .813 | .436 | 1.519 |
|  | rs16859629(2) | -.320 | .614 | .272 | 1 | .602 | .726 | .218 | 2.419 |
|  | rs16859629(3) | .109 | .141 | .591 | 1 | .442 | 1.115 | .845 | 1.470 |
|  | Constant | -1.035 | .306 | 11.435 | 1 | .001 | .355 |  |  |
| Step 2^a^ | sex(1) | 1.261 | .121 | 107.746 | 1 | .000 | 3.528 | 2.781 | 4.476 |
|  | age | .009 | .005 | 3.508 | 1 | .061 | 1.009 | 1.000 | 1.019 |
|  | smoking(1) | -1.334 | .137 | 95.126 | 1 | .000 | .264 | .202 | .345 |
|  | drinking(1) | -.753 | .154 | 23.829 | 1 | .000 | .471 | .348 | .637 |
|  | BMI(1) | .472 | .100 | 22.415 | 1 | .000 | 1.604 | 1.319 | 1.950 |
|  | Constant | -1.043 | .305 | 11.681 | 1 | .001 | .352 |  |  |
| a. Variable(s) entered on step 1: sex, age, smoking, drinking, BMI, rs16859629. | | | | | | | | | |

**Table 2b BTLA rs16859629**

| Model if Term Removed^a^ | | | | | |
| --- | --- | --- | --- | --- | --- |
| Variable | | Model Log Likelihood | Change in -2 Log Likelihood | df | Sig. of the Change |
| Step 1 | sex | -1265.425 | 114.396 | 1 | .000 |
|  | age | -1209.900 | 3.346 | 1 | .067 |
|  | smoking | -1257.790 | 99.126 | 1 | .000 |
|  | drinking | -1220.320 | 24.186 | 1 | .000 |
|  | BMI | -1219.350 | 22.245 | 1 | .000 |
|  | rs16859629 | -1208.916 | 1.378 | 3 | .711 |
| Step 2 | sex | -1266.671 | 115.509 | 1 | .000 |
|  | age | -1210.676 | 3.520 | 1 | .061 |
|  | smoking | -1258.590 | 99.347 | 1 | .000 |
|  | drinking | -1221.065 | 24.297 | 1 | .000 |
|  | BMI | -1220.193 | 22.553 | 1 | .000 |
| a. Based on conditional parameter estimates | | | | | |

**Table 3a BTLA rs2171513**

| Variables in the Equation | | | | | | | | | |
| --- | --- | --- | --- | --- | --- | --- | --- | --- | --- |
|  | | B | S.E. | Wald | df | Sig. | Exp(B) | 95% C.I.for EXP(B) | |
|  |  |  |  |  |  |  |  | Lower | Upper |
| Step 1^a^ | sex(1) | 1.258 | .122 | 107.106 | 1 | .000 | 3.519 | 2.773 | 4.466 |
|  | age | .009 | .005 | 3.472 | 1 | .062 | 1.009 | 1.000 | 1.019 |
|  | smoking(1) | -1.333 | .137 | 94.731 | 1 | .000 | .264 | .202 | .345 |
|  | drinking(1) | -.750 | .154 | 23.639 | 1 | .000 | .472 | .349 | .639 |
|  | BMI(1) | .469 | .100 | 22.070 | 1 | .000 | 1.599 | 1.314 | 1.944 |
|  | rs2171513 |  |  | .494 | 3 | .920 |  |  |  |
|  | rs2171513(1) | -.220 | .320 | .472 | 1 | .492 | .803 | .429 | 1.503 |
|  | rs2171513(2) | .028 | .250 | .013 | 1 | .910 | 1.029 | .630 | 1.681 |
|  | rs2171513(3) | -.004 | .108 | .002 | 1 | .967 | .996 | .805 | 1.231 |
|  | Constant | -1.033 | .308 | 11.259 | 1 | .001 | .356 |  |  |
| Step 2^a^ | sex(1) | 1.261 | .121 | 107.746 | 1 | .000 | 3.528 | 2.781 | 4.476 |
|  | age | .009 | .005 | 3.508 | 1 | .061 | 1.009 | 1.000 | 1.019 |
|  | smoking(1) | -1.334 | .137 | 95.126 | 1 | .000 | .264 | .202 | .345 |
|  | drinking(1) | -.753 | .154 | 23.829 | 1 | .000 | .471 | .348 | .637 |
|  | BMI(1) | .472 | .100 | 22.415 | 1 | .000 | 1.604 | 1.319 | 1.950 |
|  | Constant | -1.043 | .305 | 11.681 | 1 | .001 | .352 |  |  |
| a. Variable(s) entered on step 1: sex, age, smoking, drinking, BMI, rs2171513. | | | | | | | | | |

**Table 3b BTLA rs2171513**

| Model if Term Removed^a^ | | | | | |
| --- | --- | --- | --- | --- | --- |
| Variable | | Model Log Likelihood | Change in -2 Log Likelihood | df | Sig. of the Change |
| Step 1 | sex | -1266.057 | 114.782 | 1 | .000 |
|  | age | -1210.408 | 3.483 | 1 | .062 |
|  | smoking | -1258.129 | 98.926 | 1 | .000 |
|  | drinking | -1220.716 | 24.100 | 1 | .000 |
|  | BMI | -1219.768 | 22.204 | 1 | .000 |
|  | rs2171513 | -1208.916 | .500 | 3 | .919 |
| Step 2 | sex | -1266.671 | 115.509 | 1 | .000 |
|  | age | -1210.676 | 3.520 | 1 | .061 |
|  | smoking | -1258.590 | 99.347 | 1 | .000 |
|  | drinking | -1221.065 | 24.297 | 1 | .000 |
|  | BMI | -1220.193 | 22.553 | 1 | .000 |
| a. Based on conditional parameter estimates | | | | | |

**Table 4a BTLA rs3112270**

| Variables in the Equation | | | | | | | | | |
| --- | --- | --- | --- | --- | --- | --- | --- | --- | --- |
|  | | B | S.E. | Wald | df | Sig. | Exp(B) | 95% C.I.for EXP(B) | |
|  |  |  |  |  |  |  |  | Lower | Upper |
| Step 1^a^ | sex(1) | 1.260 | .122 | 106.930 | 1 | .000 | 3.524 | 2.775 | 4.474 |
|  | age | .009 | .005 | 3.327 | 1 | .068 | 1.009 | .999 | 1.019 |
|  | smoking(1) | -1.333 | .137 | 94.718 | 1 | .000 | .264 | .202 | .345 |
|  | drinking(1) | -.741 | .154 | 23.032 | 1 | .000 | .477 | .352 | .645 |
|  | BMI(1) | .469 | .100 | 21.964 | 1 | .000 | 1.598 | 1.314 | 1.945 |
|  | rs3112270 |  |  | 3.422 | 4 | .490 |  |  |  |
|  | rs3112270(1) | -.342 | .362 | .894 | 1 | .344 | .710 | .350 | 1.443 |
|  | rs3112270(2) | -21.278 | 22281.929 | .000 | 1 | .999 | .000 | .000 | . |
|  | rs3112270(3) | -.201 | .193 | 1.087 | 1 | .297 | .818 | .561 | 1.193 |
|  | rs3112270(4) | -.039 | .195 | .040 | 1 | .841 | .962 | .656 | 1.410 |
|  | Constant | -.902 | .350 | 6.626 | 1 | .010 | .406 |  |  |
| Step 2^a^ | sex(1) | 1.261 | .121 | 107.746 | 1 | .000 | 3.528 | 2.781 | 4.476 |
|  | age | .009 | .005 | 3.508 | 1 | .061 | 1.009 | 1.000 | 1.019 |
|  | smoking(1) | -1.334 | .137 | 95.126 | 1 | .000 | .264 | .202 | .345 |
|  | drinking(1) | -.753 | .154 | 23.829 | 1 | .000 | .471 | .348 | .637 |
|  | BMI(1) | .472 | .100 | 22.415 | 1 | .000 | 1.604 | 1.319 | 1.950 |
|  | Constant | -1.043 | .305 | 11.681 | 1 | .001 | .352 |  |  |
| a Variable(s) entered on step 1: sex, age, smoking, drinking, BMI, rs3112270. | | | | | | | | | |

**Table 4b BTLA rs3112270**

| Model if Term Removed^a^ | | | | | |
| --- | --- | --- | --- | --- | --- |
| Variable | | Model Log Likelihood | Change in -2 Log Likelihood | df | Sig. of the Change |
| Step 1 | sex | -1262.568 | 114.647 | 1 | .000 |
|  | age | -1206.913 | 3.338 | 1 | .068 |
|  | smoking | -1254.708 | 98.928 | 1 | .000 |
|  | drinking | -1216.978 | 23.468 | 1 | .000 |
|  | BMI | -1216.293 | 22.098 | 1 | .000 |
|  | rs3112270 | -1208.920 | 7.351 | 4 | .118 |
| Step 2 | sex | -1266.671 | 115.509 | 1 | .000 |
|  | age | -1210.676 | 3.520 | 1 | .061 |
|  | smoking | -1258.590 | 99.347 | 1 | .000 |
|  | drinking | -1221.065 | 24.297 | 1 | .000 |
|  | BMI | -1220.193 | 22.553 | 1 | .000 |
| a Based on conditional parameter estimates | | | | | |

**The multivariate analysis**

**Table 5a BTLA rs16859629, rs1982809, rs2171513 and rs3112270**

| Variables in the Equation | | | | | | | | | |
| --- | --- | --- | --- | --- | --- | --- | --- | --- | --- |
|  | | B | S.E. | Wald | df | Sig. | Exp(B) | 95% C.I.for EXP(B) | |
|  |  |  |  |  |  |  |  | Lower | Upper |
| Step 1^a^ | sex(1) | 1.265 | .122 | 107.260 | 1 | .000 | 3.544 | 2.789 | 4.502 |
|  | age | .009 | .005 | 3.433 | 1 | .064 | 1.009 | .999 | 1.019 |
|  | smoking(1) | -1.344 | .138 | 95.407 | 1 | .000 | .261 | .199 | .342 |
|  | drinking(1) | -.739 | .155 | 22.775 | 1 | .000 | .478 | .353 | .647 |
|  | BMI(1) | .475 | .100 | 22.367 | 1 | .000 | 1.607 | 1.320 | 1.957 |
|  | rs16859629 |  |  | 1.682 | 3 | .641 |  |  |  |
|  | rs16859629(1) | -.334 | .399 | .702 | 1 | .402 | .716 | .327 | 1.565 |
|  | rs16859629(2) | -.314 | .614 | .261 | 1 | .609 | .731 | .220 | 2.433 |
|  | rs16859629(3) | .112 | .143 | .610 | 1 | .435 | 1.119 | .844 | 1.482 |
|  | rs1982809 |  |  | 2.297 | 3 | .513 |  |  |  |
|  | rs1982809(2) | -21.549 | 27720.521 | .000 | 1 | .999 | .000 | .000 | . |
|  | rs1982809(3) | -.135 | .248 | .297 | 1 | .586 | .874 | .538 | 1.420 |
|  | rs1982809(4) | .142 | .130 | 1.202 | 1 | .273 | 1.153 | .894 | 1.488 |
|  | rs2171513 |  |  | .016 | 2 | .992 |  |  |  |
|  | rs2171513(2) | .032 | .255 | .016 | 1 | .900 | 1.032 | .626 | 1.703 |
|  | rs2171513(3) | .003 | .111 | .001 | 1 | .976 | 1.003 | .808 | 1.246 |
|  | rs3112270 |  |  | 1.033 | 3 | .793 |  |  |  |
|  | rs3112270(2) | -21.261 | 22317.956 | .000 | 1 | .999 | .000 | .000 | . |
|  | rs3112270(3) | -.226 | .241 | .880 | 1 | .348 | .798 | .497 | 1.280 |
|  | rs3112270(4) | -.132 | .223 | .349 | 1 | .554 | .876 | .566 | 1.358 |
|  | Constant | -.921 | .392 | 5.535 | 1 | .019 | .398 |  |  |
| Step 2^a^ | sex(1) | 1.265 | .122 | 107.292 | 1 | .000 | 3.543 | 2.789 | 4.501 |
|  | age | .009 | .005 | 3.430 | 1 | .064 | 1.009 | .999 | 1.019 |
|  | smoking(1) | -1.344 | .137 | 95.597 | 1 | .000 | .261 | .199 | .341 |
|  | drinking(1) | -.739 | .155 | 22.832 | 1 | .000 | .477 | .353 | .647 |
|  | BMI(1) | .475 | .100 | 22.370 | 1 | .000 | 1.607 | 1.320 | 1.957 |
|  | rs16859629 |  |  | 1.696 | 3 | .638 |  |  |  |
|  | rs16859629(1) | -.339 | .394 | .741 | 1 | .389 | .712 | .329 | 1.543 |
|  | rs16859629(2) | -.316 | .612 | .266 | 1 | .606 | .729 | .219 | 2.421 |
|  | rs16859629(3) | .110 | .142 | .601 | 1 | .438 | 1.117 | .845 | 1.476 |
|  | rs1982809 |  |  | 2.291 | 3 | .514 |  |  |  |
|  | rs1982809(2) | -21.551 | 27719.041 | .000 | 1 | .999 | .000 | .000 | . |
|  | rs1982809(3) | -.139 | .245 | .319 | 1 | .572 | .871 | .538 | 1.408 |
|  | rs1982809(4) | .140 | .128 | 1.201 | 1 | .273 | 1.150 | .895 | 1.478 |
|  | rs3112270 |  |  | 1.089 | 3 | .780 |  |  |  |
|  | rs3112270(2) | -21.263 | 22322.261 | .000 | 1 | .999 | .000 | .000 | . |
|  | rs3112270(3) | -.229 | .240 | .908 | 1 | .341 | .796 | .497 | 1.273 |
|  | rs3112270(4) | -.132 | .223 | .350 | 1 | .554 | .876 | .566 | 1.357 |
|  | Constant | -.916 | .386 | 5.624 | 1 | .018 | .400 |  |  |
| Step 3^a^ | sex(1) | 1.271 | .122 | 108.562 | 1 | .000 | 3.564 | 2.806 | 4.527 |
|  | age | .010 | .005 | 3.619 | 1 | .057 | 1.010 | 1.000 | 1.019 |
|  | smoking(1) | -1.344 | .137 | 95.749 | 1 | .000 | .261 | .199 | .341 |
|  | drinking(1) | -.742 | .155 | 23.013 | 1 | .000 | .476 | .352 | .645 |
|  | BMI(1) | .477 | .100 | 22.614 | 1 | .000 | 1.611 | 1.323 | 1.960 |
|  | rs1982809 |  |  | 2.310 | 3 | .511 |  |  |  |
|  | rs1982809(2) | -21.548 | 27746.219 | .000 | 1 | .999 | .000 | .000 | . |
|  | rs1982809(3) | -.039 | .226 | .029 | 1 | .864 | .962 | .618 | 1.498 |
|  | rs1982809(4) | .171 | .124 | 1.917 | 1 | .166 | 1.187 | .931 | 1.513 |
|  | rs3112270 |  |  | .501 | 3 | .919 |  |  |  |
|  | rs3112270(2) | -21.140 | 22331.085 | .000 | 1 | .999 | .000 | .000 | . |
|  | rs3112270(3) | -.106 | .195 | .296 | 1 | .586 | .899 | .614 | 1.317 |
|  | rs3112270(4) | -.029 | .189 | .023 | 1 | .879 | .972 | .670 | 1.408 |
|  | Constant | -1.050 | .355 | 8.757 | 1 | .003 | .350 |  |  |
| Step 4^a^ | sex(1) | 1.267 | .122 | 108.243 | 1 | .000 | 3.551 | 2.797 | 4.508 |
|  | age | .010 | .005 | 3.745 | 1 | .053 | 1.010 | 1.000 | 1.020 |
|  | smoking(1) | -1.341 | .137 | 95.552 | 1 | .000 | .262 | .200 | .342 |
|  | drinking(1) | -.744 | .154 | 23.217 | 1 | .000 | .475 | .351 | .643 |
|  | BMI(1) | .478 | .100 | 22.830 | 1 | .000 | 1.613 | 1.326 | 1.962 |
|  | rs1982809 |  |  | 4.567 | 3 | .206 |  |  |  |
|  | rs1982809(2) | -21.516 | 27845.266 | .000 | 1 | .999 | .000 | .000 | . |
|  | rs1982809(3) | .011 | .196 | .003 | 1 | .957 | 1.011 | .688 | 1.485 |
|  | **rs1982809(4)** | **.217** | **.103** | **4.413** | **1** | **.036** | 1.243 | 1.015 | 1.522 |
|  | Constant | -1.146 | .310 | 13.644 | 1 | .000 | .318 |  |  |
| a. Variable(s) entered on step 1: sex, age, smoking, drinking, BMI, rs16859629, rs1982809, rs2171513, rs3112270. | | | | | | | | | |

**Table 5b BTLA rs16859629, rs1982809, rs2171513 and rs3112270**

| Model if Term Removed^a^ | | | | | |
| --- | --- | --- | --- | --- | --- |
| Variable | | Model Log Likelihood | Change in -2 Log Likelihood | df | Sig. of the Change |
| Step 1 | sex | -1259.393 | 115.087 | 1 | .000 |
|  | age | -1203.571 | 3.444 | 1 | .063 |
|  | smoking | -1251.715 | 99.732 | 1 | .000 |
|  | drinking | -1213.449 | 23.200 | 1 | .000 |
|  | BMI | -1213.105 | 22.511 | 1 | .000 |
|  | rs16859629 | -1202.696 | 1.693 | 3 | .639 |
|  | rs1982809 | -1204.813 | 5.927 | 3 | .115 |
|  | rs2171513 | -1201.857 | .016 | 2 | .992 |
|  | rs3112270 | -1204.288 | 4.877 | 3 | .181 |
| Step 2 | sex | -1259.416 | 115.117 | 1 | .000 |
|  | age | -1203.578 | 3.441 | 1 | .064 |
|  | smoking | -1251.823 | 99.932 | 1 | .000 |
|  | drinking | -1213.486 | 23.258 | 1 | .000 |
|  | BMI | -1213.114 | 22.514 | 1 | .000 |
|  | rs16859629 | -1202.711 | 1.708 | 3 | .635 |
|  | rs1982809 | -1204.821 | 5.927 | 3 | .115 |
|  | rs3112270 | -1204.330 | 4.945 | 3 | .176 |
| Step 3 | sex | -1261.004 | 116.586 | 1 | .000 |
|  | age | -1204.527 | 3.631 | 1 | .057 |
|  | smoking | -1252.754 | 100.086 | 1 | .000 |
|  | drinking | -1214.434 | 23.447 | 1 | .000 |
|  | BMI | -1214.092 | 22.762 | 1 | .000 |
|  | rs1982809 | -1205.701 | 5.980 | 3 | .113 |
|  | rs3112270 | -1204.838 | 4.254 | 3 | .235 |
| Step 4 | sex | -1262.908 | 116.160 | 1 | .000 |
|  | age | -1206.708 | 3.759 | 1 | .053 |
|  | smoking | -1254.753 | 99.850 | 1 | .000 |
|  | drinking | -1216.659 | 23.662 | 1 | .000 |
|  | BMI | -1216.318 | 22.979 | 1 | .000 |
|  | rs1982809 | -1208.920 | 8.182 | 3 | .042 |
| a. Based on conditional parameter estimates | | | | | |
